# Combined achalasia and cricopharyngeal achalasia in a patient with type 1 myotonic dystrophy: a case report 

**Published:** 2020

**Authors:** Sami Ghazaleh, Christian Nehme, Yasmin Khader, Syed Hasan, Ali Nawras

**Affiliations:** 1 *Department of Internal Medicine, University of Toledo Medical Center, Toledo, Ohio, USA*; 2 *Department of Gastroenterology, University of Toledo Medical Center, Toledo, Ohio, USA *

**Keywords:** Esophageal achalasia, Dysphagia, Myotonic dystrophy

## Abstract

Type 1 myotonic dystrophy (MD) is a rare inherited disease which presents with skeletal muscle weakness and myotonia. Involvement of smooth muscles is also common and mainly manifests in the gastrointestinal tract. We report a case of type 1 MD who presented with dysphagia and was found to have unique esophageal manometry findings. A 57-year-old male patient presented with dysphagia for the last few months. Past medical history was significant for type 1 myotonic muscular dystrophy, gastroesophageal reflux disease, diaphragmatic paralysis, and obstructive sleep apnea. Both his father and brother died in their 50s because of unclear respiratory problems. He was a former smoker and did not drink alcohol. Review of systems was unremarkable. His neurological examination was significant for bilateral facial muscle weakness and mild ptosis. He had atrophy and weakness of the distal upper and lower extremities. Deep tendon reflexes were absent. Upper endoscopy and 24-hour esophageal pH testing were non-diagnostic. Finally, esophageal manometry revealed elevated lower esophageal sphincter (LES) pressure, elevated upper esophageal sphincter (UES) pressure, and very week peristalsis of the esophageal body. Esophageal involvement is common in type 1 MD manifesting with dysfunction of UES, esophageal body, and LES. Manometry usually describes a reduced resting tone of the UES and LES. The patient had elevated LES pressure and week peristalsis of the esophageal body consistent with achalasia. He also had an elevated UES pressure consistent with cricopharyngeal achalasia. This is the opposite of what is expected in type 1 MD.

## Introduction

 The esophagus is a muscular tube that connects the pharynx to the stomach (1). The lower esophageal sphincter (LES) is a segment of the muscular layer of the distal esophagus which prevents stomach contents and acid from travelling back to the esophagus. During swallowing, it relaxes to allow food to travel from the esophagus to the stomach. The upper esophageal sphincter (UES) is a muscular structure which is mainly composed of the cricopharyngeus muscle. When it is closed, it prevents esophageal air insufflation during inspiration and prevents laryngeal reflux during esophageal peristalsis. It relaxes to allow passage of food or air during swallowing or emesis (2). 

Achalasia is an uncommon disorder caused by degeneration of the inhibitory ganglion cells in the myenteric plexus of the esophagus (3). This leads to failure of the lower esophageal sphincter to relax during swallowing in addition to aperistalsis in the esophageal body. Patients usually complain of dysphagia, regurgitation, chest pain, and weight loss. Dysphagia tend to progress over time and often involves both solids and liquids (4). 

Cricopharyngeal achalasia or cricopharyngeal dysfunction are terms that describe disorders that involve the cricopharyngeus muscle or the UES. Patients usually complain of difficulty initiating a swallow which can lead to nasopharyngeal regurgitation, choking, and aspiration (5). Several conditions can cause cricopharyngeal achalasia though some patients can have primary cricopharyngeal dysfunction (6). Neurological and myopathic disorders frequently associated with cricopharyngeal achalasia include stroke (7), motor neuron disease (8), Parkinson’s disease (9), myasthenia gravis (10), and myotonic dystrophy (11).

We report a case of type 1 myotonic dystrophy (MD) who presented with progressive dysphagia. He was found to have achalasia with the classic findings of elevated LES pressure and aperistalsis in the esophageal body. In addition, he had cricopharyngeal achalasia with elevated UES pressure.

## Case Report

A 57-year-old Caucasian male patient presented to the gastroenterology clinic with progressive dysphagia to solids and liquids for the last few months. The patient had a history of type 1 myotonic muscular dystrophy diagnosed at the age of 34, gastroesophageal reflux disease (GERD), diaphragmatic paralysis, and obstructive sleep apnea. He complained of occasional heartburn, but denied weight loss, anorexia, chest pain, regurgitation, cough, early satiety, abdominal pain, nausea, vomiting, diarrhea, hematochezia, or melena. Last upper endoscopy 5 years ago revealed a small hiatal hernia and a normal esophagus, stomach, and duodenum. His only home medication was a daily 40 mg tablet of omeprazole. Past surgical history was significant for bilateral cataract surgery. Both his father and brother died in their 50s because of unclear respiratory problems, and there was no family history of malignancies. He was a former smoker and did not drink alcohol. 

On physical examination, the patient was alert, oriented, and in no acute distress. Vital signs demonstrated a temperature of 36.8° C, blood pressure of 126/85 mmHg, heart rate of 90 beats per minute, respiratory rate of 16 breaths per minute, and O2 saturation of 97% on room air. Cardiovascular, lung, abdominal, and skin exams were unremarkable. 

Neurological examination was significant for bilateral facial muscle weakness and mild ptosis. His other cranial nerves were intact. He had atrophy and weakness of the distal upper and lower extremities. Deep tendon reflexes were absent. Sensory, cerebellar, and gait exams were unremarkable.

Laboratory workup including complete blood count (CBC) and basic metabolic panel (BMP) were within normal limits. An upper endoscopy was performed and showed mild hyperemia of the stomach, but a normal esophagus and no hiatal hernia. Esophageal biopsies were obtained from the upper and lower esophagus which were normal and showed no evidence of eosinophilic esophagitis.

After two weeks of being off omeprazole, the patient underwent a 24-hour esophageal pH testing which showed no significant acid reflux. Finally, he underwent esophageal manometry which revealed elevated LES pressure, elevated UES pressure, and very week peristalsis of the esophageal body. 

## Discussion

Type 1 MD is a rare autosomal dominant disease which presents with skeletal muscle weakness, myotonia, cardiac abnormalities, respiratory dysfunction, sleep disturbances, and infertility (12). Involvement of smooth muscles is common and mainly manifests in the gastrointestinal (GI) tract. Common GI manifestations include dysphagia, GERD, constipation, and gallbladder problems (11, 13). Type 1 MD frequently affects the esophagus manifesting with dysphagia which increases the risk of aspiration. 

Manometry is particularly useful in detecting esophageal motility problems. The literature is consistent on the effect of type 1 MD on the UES and esophageal body pressures during swallowing. Most studies have reported a decreased UES resting pressure and a normal UES relaxation pressure. Studies have also demonstrated a reduced amplitude of the esophageal peristaltic waves (14-20). Involvement of the LES is less clear as some studies have found a reduced LES resting pressure and a higher risk of GERD while others failed to demonstrate an abnormal LES pressure (15, 16, 18, 21). 

Histopathologic examination of the esophagus can provide clues on the underlying mechanism of esophageal dysfunction, but the pathophysiology behind this pattern of esophageal dysfunction remains unknown. The upper esophagus mainly contains striated muscles while the lower esophagus has smooth muscles. In patients with type 1 MD, muscle biopsies of the upper esophagus have shown abnormal skeletal muscle fibers with variations in size, atrophy, and necrosis. Biopsies of the lower esophagus, however, have not shown abnormalities of the smooth muscles even in studies where the LES pressure was reduced (18).

Our patient, however, had elevated UES and LES pressures and week peristalsis of the esophageal body. This is unusual in type 1 MD where the commonly seen picture is a decreased resting tone of the UES and LES. It is important to note that increased UES pressure has been reported in patients with achalasia, so our patient might have had a primary esophageal achalasia with combined increase in the tones of LES and UES (22). As we poorly understand the pathophysiology of esophageal involvement in type 1 MD, the exact mechanism of esophageal dysfunction in our patient remains unclear. 

Esophageal involvement is common in type 1 MD manifesting with dysfunction of UES, esophageal body, and LES. The exact mechanism is still poorly understood, but manometry usually describes a reduced resting tone of the UES and LES. Our patient, however, had elevated UES and LES pressures which is in contrast to what is expected in type 1 MD. Further studies are recommended to investigate the pathophysiology and histopathology of esophageal dysfunction in patients with MD.

## Conflict of interests

The authors declare that they have no conflict of interest.
